# Clarifying the roles of schizotypy and psychopathic traits in lexical decision performance

**DOI:** 10.1016/j.scog.2021.100224

**Published:** 2021-11-16

**Authors:** Martina Vanova, Luke Aldridge-Waddon, Ben Jennings, Leonie Elbers, Ignazio Puzzo, Veena Kumari

**Affiliations:** a Division of Psychology, Department of Life Sciences, & Centre for Cognitive Neuroscience, College of Health, Medicine and Life Sciences, Brunel University London, UK; b Department of Psychology, University of Wuppertal, Germany

**Keywords:** Schizotypy, Psychopathy, Impulsivity, Lexical decision task, Lexical recognition, Reading skills

## Abstract

**Introduction:**

Some studies suggest that lexical recognition is impaired in people with schizophrenia, psychopathy and/or antisocial personality disorders, but not affective disorders. We examined the extent to which various traits dimensionally linked to one or more of these disorders are associated with lexical recognition performance in the general population.

**Methods:**

Seventy-eight healthy English-speaking participants completed self-report measures of schizotypy, psychopathy, impulsivity, depression, anxiety and stress. All participants were assessed on a one-choice variant of a lexical decision task (LDT).

**Results:**

Meanness and Boldness traits of psychopathy (Triarchic Psychopathy Measure), and positive schizotypy (Unusual Experiences, Oxford-Liverpool Inventory of Feelings and Experiences) were associated with poor word-nonword accuracy, and predicted a significant amount of unique variance (Meanness, 12%; Boldness, 4.8%; Positive Schizotypy, 4.4%; total 21%) in performance. Higher motor impulsivity predicted 30% of the variance in low-frequency words recognition accuracy, but only in non-native English speakers. Affective traits were not associated with LDT performance.

**Conclusion:**

Psychopathic traits show stronger negative associations with lexical recognition performance than schizotypal traits, and impulsivity may differently influence lexical decision performance in native and non-native speakers. Further studies are needed to replicate these findings, especially the influence of language familiarity in the impulsivity-performance relationship, and to clarify the influence of corresponding symptom dimensions in lexical recognition abilities, taking language familiarity, migration status, and comorbidity into account, in people with schizophrenia, psychopathy, and/or antisocial personality disorders.

## 1 Introduction

Reading begins with the recognition or decoding of words and comparison of the written-read entries with the person's vocabulary in memory (Gough and Tunmer, 1986; James and Oberle, 2012). According to the Dual Route Cascaded model, words can be identified by following the sublexical or lexical pathway (Coltheart et al., 2001). The sublexical pathway recognises words by decoding letters into sounds, putting them together, and comparing the outcome with existing mental vocabulary entries. This pathway engages phonological processing, orthography, and semantic skills, and is used in the recognition of unfamiliar words (often low-frequency) and nonwords. In the lexical pathway, a familiar word (often high-frequency) is recognised as a whole, triggering automatic mental representation (Balota and Yap, 2006; Coltheart et al., 2001). Lexical recognition is a good indicator of overall reading proficiency, especially in bilingual individuals (Harrington, 2006; Park et al., 2012), and typically assessed using variants of the lexical decision task (LDT) requiring participants to identify a string of letters as a word or nonword (Meyer and Schvaneveldt, 1971).

A recent meta-analysis (Vanova et al., 2021) revealed significant deficits in reading skills in schizophrenia, personality disorders and/or psychopathy, but not in affective disorders. In the context of LDT, individuals with schizophrenia showed poorer word-nonword recognition and longer reaction times (RTs) than controls in some (Hokama et al., 2003), but not all studies (Natsubori et al., 2014; Tan et al., 2016b). The relationship between schizotypy, a potential vulnerability factor for schizophrenia (Lenzenweger, 2018), and LDT performance is unclear (Schofield and Mohr, 2014), with reports of similar performance in groups with high and low schizotypy (Park and Waldie, 2017), and no significant dimensional relationships between schizotypy and LDT performance (Carlin and Lindell, 2015; Tan et al., 2016a) though Cognitive Disorganisation aspect of schizotypy did predict nonword errors in one study (Tan et al., 2016a).

Psychopathy has been associated with poorer reading skills in forensic and community samples (Vanova et al., 2021). Higher impulsive-antisocial psychopathy scores correlate with poorer overall word-nonword recognition (Heritage and Benning, 2013; Lorenz and Newman, 2002), and slower RTs, especially in forensic samples (Kiehl et al., 2004; Reidy et al., 2008). Impulsivity, a core feature of multiple psychopathologies (Whiteside and Lynam, 2001), is commonly present in individuals with psychopathic traits (Weidacker et al., 2017) or schizotypy (Mason and Claridge, 2006). One study (Harmon-Jones et al., 1997) observed higher attentional and non-planning, but not motor, impulsivity to be related to poor reading comprehension and accuracy, while another study (De Pascalis et al., 2009) reported a negative influence of higher overall impulsivity on the RTs and accuracy when processing words incongruent with presented sentences. Previous research suggests intact reading skills in people with affective disorders (Vanova et al., 2021), and no effect of subclinical depression and anxiety in word-nonword recognition (Li et al., 2014; Notebaert et al., 2019; Stevens et al., 2015; White et al., 2010). However, much of the evidence for reading skills deficits in clinical populations comes from small sample studies with high heterogeneity, and rarely accounts for confounders such as medication (Wright and Woods, 2020).

The present study, therefore, examined the relationship between schizotypy, psychopathy, impulsivity, affective traits, and LDT performance, in a general population sample. Based on previous findings (Vanova et al., 2021), we hypothesised that higher schizotypy, psychopathy, and impulsivity will be associated with lower LDT performance. Furthermore, we examined the common and unique contribution of schizotypy, psychopathy and/or impulsivity to LDT performance and explored the role of language familiarity (native versus non-native speakers) in these associations.

## 2 Methods

### 2.1 Participants

Seventy-eight healthy adults with sufficient written and verbal command of the English language, normal/corrected-to-normal vision and hearing, no self-reported incidence of psychiatric/neurological illness, and no serious criminal history participated. The study was approved by the university research ethics committee. Participants provided written informed consent and were compensated for their time.

### 2.2 Materials

#### 2.2.1 Self-report measures of psychopathology-related traits

Schizotypy was assessed using the Oxford-Liverpool Inventory of Feelings and Experiences (O-LIFE; 150 items; subscales: Unusual Experiences, Cognitive Disorganisation, Introvertive Anhedonia, Impulsive Nonconformity) (Mason and Claridge, 2006). Psychopathy was assessed using the Self-Report Psychopathy Scale–Short Form (SRP-4-SFl; 29 items; subscales: Interpersonal, Affective, Lifestyle, Antisocial) (Paulhus et al., 2016) and Triarchic Psychopathy Measure (TriPM; 58 items; subscales: Boldness, Meanness, Disinhibition) (Patrick et al., 2009). Impulsivity was assessed using the Barratt Impulsiveness Scale (BIS-11; 30 items; subscales: Attention, Cognitive Instability, Motor, Perseverance, Self-Control, Cognitive Complexity) (Patton et al., 1995) and Impulsive Behavior Scale-Short (S-UPPS-P; 20 items; Negative Urgency, Positive Urgency, Lack of Premeditation, Lack of Perseverance, Sensation Seeking) (Whiteside et al., 2005). Affective traits were assessed using the Depression, Anxiety, and Stress Scale (DASS-21, 21 items) (Lovibond and Lovibond, 1995). All measures were administered using Qualtrics^XM^ (Qualtrics LLC, 2005).

#### 2.2.2 Lexical decision task (LDT)

**Fig. 1:**
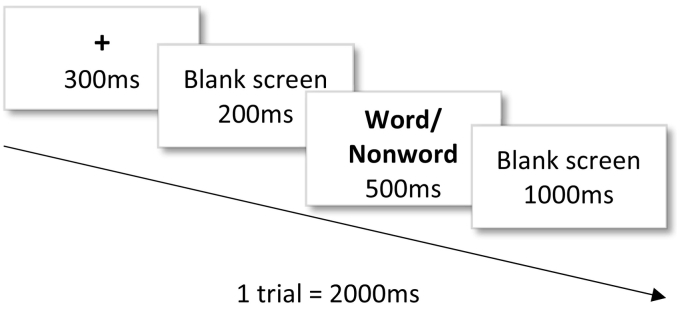
Lexical decision task trial.

The task was administered using Presentation® Software (version 21.1) (Neurobehavioral Systems Inc., 2018). Participants were presented with 120 stimuli (5–6 letters long) consisting of 60 English words from the frequency list of the British National Corpus (Leech et al., 2001) and 60 nonwords from the ARC Database (Rastle et al., 2002). The word list consisted of 30 high-frequency (2900–3000 occurrences per million words) and 30 low-frequency word lemmas (10–11 /million), counterbalanced per word category (adjectives, verbs, nouns). The nonword list included 30 real nonwords (letter strings not existing in the English language and not resembling any existing word, e.g., *youns*, *cimes*) and 30 pseudohomophones (nonwords pronounced as recognisable words but spelt incorrectly, e.g., *hense* [hence]). The nonword list was counterbalanced in the summed frequency of nonword neighbours, which is an indicator of similarity with other nonwords (high-frequency: 300–700/million; low-frequency: 1–10/million). The neighbourhood size for all nonwords was 1, representing the number of words that can be derived by changing one letter. Each trial consisted of a 300 ms fixation cross, a 200 ms blank screen, a 500 ms main stimulus (word/nonword), and a 1000 ms (blank screen) response period (Fig. 1).

Participants were asked to respond with a button press when presented with a valid English word and make no response to nonwords. The instructions were presented before a practice session (with feedback) consisting of 16 stimuli (50% words). Performance was indexed by response accuracy (RA) and speed (RTs). RAs for words were examined as the number of correct button-presses and for nonwords as correct withdrawals. Overall performance was calculated as the number of correctly identified words plus nonwords. RTs (in ms) were assessed for correct responses to high and low-frequency words, and incorrect responses to pseudohomophones and real nonwords.

### 2.3 Statistical analyses

All analyses were performed using IBM SPSS Statistics, V26.0 (IBM Corp., 2019), with p ≤ 0.05. All variables were first assessed for normality, and those with significant skewness or kurtosis were normalised by replacing outliers with mean value ±2SD for each variable (Field, 2009) (Table 1, Table 2).

**Table 1:** Descriptive statistics for self-report psychopathology measures (N = 78).

	Mean (SD)	Observed min	Observed max	Maximum possible score
O-LIFE unusual experiences	10.4 (6.24)	0	25	30
O-LIFE cognitive distortions	13.3 (5.46)	0	24	24
O-LIFE introvertive anhedonia^a^	7.42 (4.63)	0	22	27
O-LIFE impulsive nonconformity	8.91 (3.30)	3	17	23
SRP-4-SF interpersonal^a^	13.8 (4.93)	7	28	35
SRP-4-SF affective	14.2 (4.68)	7	30	35
SRP-4-SF lifestyle	15.8 (5.00)	7	29	35
SRP-4-SF antisocial^a^	9.99 (2.24)	8	22	40
TriPM boldness	27.2 (8.26)	10	46	76
TriPM disinhibition^a^	14.8 (7.70)	1	34	80
TriPM meanness	13.2 (6.18)	1	27	76
BIS-11 attention^a^	10.8 (2.78)	6	20	20
BIS-11 cognitive instability	6.31 (2.24)	3	12	12
BIS-11 motor	14.4 (3.26)	7	22	28
BIS-11 perseverance^a^	7.15 (1.83)	3	14	16
BIS-11 self-control	13.2 (3.68)	7	21	24
BIS-11 cognitive complexity	11.2 (2.26)	6	16	20
S-UPPS-P negative urgency	8.77 (2.82)	4	15	16
S-UPPS-P lack of perseverance	7.46 (1.79)	4	11	16
S-UPPS-P lack of premeditation	7.36 (2.27)	4	12	16
S-UPPS-P sensation seeking	10.7 (2.86)	4	16	16
S-UPPS-P positive urgency	8.01 (2.71)	4	15	16
DASS-21 depression^a^	13 (4.71)	7	28	28
DASS-21 anxiety^a^	13.1 (4.54)	7	26	28
DASS-21 stress	14.7 (4.14)	7	24	28

a Normalised by replacing outliers (all had scores above mean + 2SD; no >6 people for any variable) with Mean ± 2SD. O-LIFE = Oxford-Liverpool Inventory of Feelings and Experiences; SRP-4-SF = Self-Report Psychopathy Scale – Short Form; TriPM = Triarchic Psychopathy Measure; BIS-11 = Barratt Impulsiveness Scale; S-UPPS-P = Impulsive Behavior Scale, Short Version; DASS-21 = Depression, Anxiety, and Stress Scale.

**Table 2:** Descriptive statistics for task performance for the entire sample and differences between native and non-native speakers.

	Entire Sample (N = 78)			Native speakers (n = 42)	Non-native speakers (n = 36)	Group differences (native versus non-native speakers)		
	Mean (SD)	Range	Maximum possible score	Mean (SD)	Mean (SD)	*t* (df = 76)	p	Cohen's *d*
Overall performance^a^	105.10 (7.35)	77–118	120	107.60 (5.70)	102.20 (8.04)	3.360	**<0.001**^⁎⁎⁎^	0.784
Correct words high-frequency^a^	29.81 (0.47)	25–30	30	29.90 (0.30)	29.70 (0.59)	1.876	0.053	0.446
Correct words low-frequency^a^	27.09 (2.16)	15–30	30	27.51 (1.98)	26.61 (2.28)	1.867	0.066	0.424
Correct pseudohomophones^a^	24.21 (3.55)	13–29	30	25.29 (2.78)	22.94 (3.95)	3.000	**0.004**^⁎⁎^	0.700
Correct real nonwords^a^	24.17 (3.52)	13–29	30	25.02 (2.75)	23.18 (4.07)	2.307	**0.024**^⁎^	0.539
Correct words high-frequency RT	417.67 (35.02)	327–496	1000	415.87 (35.99)	419.78 (34.26)	0.488	0.627	0.111
Correct words low-frequency RT	478.93 (48.80)	357–621	1000	473.50 (50.96)	485.26 (46.07)	1.062	0.292	0.241
Incorrect pseudohomophones RT	449.08 (82.51)	297–635	1000	453.07 (83.87)	444.28 (81.84)	0.459	0.648	0.104
Incorrect real nonwords RT	429.58 (68.95)	293–579	1000	420.04 (56.33)	440.70 (80.66)	1.290	0.202	0.301

* p < 0.05; ** p < 0.01; *** p < 0.001*.* Significant differences are in **bold**.

a Normalised by replacing outliers (all had scores below mean-2SD; no more than six outliers for any variable) with mean-2SD.

Differences between native and non-native speakers in categorical variables were explored using Chi-Square, and in continuous variables using independent sample *t*-tests. Performance accuracy was analysed using a 4 (Stimulus-Type) × 2 (Sex) × 2 (Language) analysis of variance (ANOVA) with Stimulus-Type (high-frequency words, low-frequency words, pseudohomophones, real nonwords) as a within-subject factor, and Sex (males, females) and Language (native speakers, non-native speakers) as the between-subject factors. RTs to correct high and low-frequency words and incorrect pseudohomophones and real nonwords were analysed (separately) by 2 (Stimulus-Type: high and low-frequency words/pseudohomophones, real nonwords) × 2 (Sex) × 2 (Language) ANOVA with Stimulus-Type as a within-subject variable. The Greenhouse-Geisser correction was applied where Mauchly's Test indicated a significant sphericity violation.

Spearman's rank-order correlation coefficients (*r*_s_) were used to examine psychopathology-LDT performance associations, first, across the whole sample, and then separately in native and non-native speakers, followed by the strength of the correlations in these two groups formally compared using Fisher's z transformation. Correction for multiple correlations was not applied because we wished to comprehensively explore the influence of all relevant trait dimensions, and expected, at best, small-to-moderate correlations. The overall LDT performance and RTs for incorrect real nonwords were associated, as shown in Table 3, with two or more traits (inter-relationships among various traits presented in Supplementary Table 2) and thus, analysed further using linear regression ‘Stepwise’ method. This method determines the final model based on a process of selecting/eliminating predictors one at a time depending on the outcome of the *t*-tests for the slope parameters, (i.e., partial F-tests) and the amount of shared and unique variance explained by these predictors.

**Table 3:** Spearman rank-order correlations (*r*_*s*_) between LDT performance and schizotypy and psychopathy measures in the entire sample (N = 78).

Accuracy Measure	Overall performance accuracy	Correct words high-frequency	Correct words low-frequency	Correct pseudo-homophones	Correct real nonwords	Correct words high-frequency RTs	Correct words low-frequency RTs	Incorrect pseudo-homophones RTs	Incorrect real nonwords RTs
	*r_s_* (p)	*r*_*s*_ (p)	*r*_*s*_ (p)	*r*_*s*_ (p)	*r*_*s*_ (p)	*r*_*s*_ (p)	*r*_*s*_ (p)	*r*_*s*_ (p)	r_s_ (p)
O-LIFE unusual experiences	−0.248^⁎^ (0.028)	−0.130 (0.256)	−0.204 (0.073)	−0.196 (0.086)	−0.194 (0.089)	−0.029 (0.803)	−0.020 (0.860)	−0.040 (0.729)	0.019 (0.865)
O-LIFE cognitive distortions	0.035 (0.763)	0.022 (0.845)	−0.025 (0.827)	−0.007 (0.950)	0.077 (0.501)	−0.019 (0.870)	0.072 (0.529)	0.006 (0.956)	0.062 (0.591)
O-LIFE introvertive anhedonia	−0.054 (0.639)	0.002 (0.984)	−0.117 (0.309)	−0.081 (0.479)	0.022 (0.851)	−0.071 (0.538)	−0.049 (0.667)	−0.083 (0.472)	−0.057 (0.618)
O-LIFE impulsive nonconformity	−0.125 (0.277)	−0.081 (0.478)	0.022 (0.846)	−0.120 (0.295)	−0.108 (0.347)	−0.077 (0.504)	−0.155 (0.176)	−0.007 (0.954)	−0.028 (0.809)
SRP-4-SF interpersonal	−0.139 (0.223)	−0.020 (0.859)	0.066 (0.566)	**−0.244**^⁎^ (0.032)	−0.048 (0.677)	−0.003 (0.976)	−0.122 (0.288)	−0.014 (0.905)	0.026 (0.822)
SRP-4-SF affective	**−0.247**^⁎^ (0.029)	−0.011 (0.924)	−0.046 (0.690)	−0.265* (0.019)	−0.212 (0.062)	−0.074 (0.522)	−0.186 (0.103)	−0.133 (0.246)	−0.089 (0.436)
SRP-4-SF lifestyle	−0.222 (0.051)	−0.107 (0.350)	−0.087 (0.446)	−0.206 (0.070)	−0.178 (0.120)	0.003 (0.983)	−0.074 (0.518)	−0.038 (0.740)	−0.005 (0.968)
SRP-4-SF antisocial	**−0.318**^⁎⁎^ (0.005)	**−0.336**^⁎⁎^ (0.003)	**−0.244**^⁎^ (0.032)	**−0.264**^⁎^ (0.020)	−0.185 (0.105)	0.041 (0.723)	−0.049 (0.673)	−0.254* (0.025)	−0.189 (0.097)
TriPM boldness	**−0.242**^⁎^ (0.033)	−0.073 (0.526)	−0.118 (0.302)	−0.061 (0.594)	**−0.320**^⁎⁎^ (0.004)	−0.068 (0.554)	−0.205 (0.072)	−0.135 (0.237)	−0.294** (0.009)
TriPM disinhibition	−0.198 (0.082)	−0.105 (0.359)	−0.151 (0.187)	−0.203 (0.074)	−0.151 (0.188)	0.050 (0.663)	−0.079 (0.492)	−0.136 (0.235)	−0.124 (0.278)
TriPM meanness	**−0.318**^⁎⁎^ (0.005)	−0.121 (0.291)	−0.115 (0.315)	**−0.257**^⁎^ (0.023)	**−0.272**^⁎^ (0.016)	0.015 (0.899)	−0.182 (0.110)	−0.050 (0.665)	−0.055 (0.632)
BIS-11 attention	0.016 (0.890)	−0.113 (0.324)	0.214 (0.060)	−0.037 (0.746)	−0.124 (0.281)	−0.092 (0.424)	−0.166 (0.146)	0.025 (0.831)	−0.112 (0.331)
BIS-11 cognitive instability	0.024 (0.838)	−0.006 (0.960)	0.212 (0.063)	−0.039 (0.734)	−0.053 (0.645)	−0.043 (0.711)	−0.081 (0.481)	0.055 (0.633)	0.078 (0.495)
BIS-11 motor	−0.214 (0.060)	−0.211 (0.064)	**−0.281**^⁎^ (0.013)	−0.096 (0.403)	−0.157 (0.169)	0.088 (0.444)	−0.092 (0.423)	−0.105 (0.360)	−0.145 (0.204)
BIS-11 perseverance	0.018 (0.872)	0.105 (0.360)	0.082 (0.476)	0.058 (0.611)	−0.085 (0.457)	0.128 (0.265)	0.124 (0.279)	0.214 (0.060)	**0.239**^⁎^ (0.035)
BIS-11 self-control	−0.134 (0.242)	−0.045 (0.695)	**−0.284**^⁎^ (0.012)	−0.053 (0.647)	−0.055 (0.634)	0.051 (0.655)	0.009 (0.935)	0.001 (0.992)	−0.032 (0.778)
BIS-11 cognitive complexity	0.100 (0.382)	−0.109 (0.340)	−0.171 (0.133)	0.141 (0.219)	0.133 (0.247)	0.060 (0.600)	−0.049 (0.671)	0.031 (0.785)	−0.040 (0.729)
S-UPPS-P negative urgency	−0.121 (0.290)	−0.034 (0.765)	−0.077 (0.502)	−0.098 (0.393)	−0.103 (0.371)	0.006 (0.957)	0.052 (0.649)	−0.073 (0.525)	0.041 (0.721)
S-UPPS-P lack of perseverance	0.071 (0.539)	0.164 (0.151)	−0.084 (0.465)	0.026 (0.819)	0.196 (0.086)	0.054 (0.636)	0.117 (0.306)	0.161 (0.160)	0.199 (0.080)
S-UPPS-P lack of Premeditation	−0.047 (0.685)	−0.104 (0.365)	−0.122 (0.288)	−0.054 (0.638)	0.029 (0.798)	−0.050 (0.666)	−0.092 (0.424)	−0.043 (0.710)	−0.068 (0.555)
S-UPPS-P sensation seeking	**−0.293**^⁎⁎^ (0.009)	−0.082 (0.477)	−0.196 (0.086)	−0.118 (0.305)	**−0.324**^⁎⁎^ (0.004)	−0.099 (0.386)	−0.138 (0.227)	−0.038 (0.744)	−0.159 (0.165)
S-UPPS-P positive urgency	−0.203 (0.074)	−0.155 (0.175)	**−0.226**^⁎^ (0.047)	−0.125 (0.277)	−0.160 (0.162)	0.034 (0.767)	−0.085 (0.458)	−0.089 (0.437)	−0.149 (0.193)
DASS-21 depression	−0.061 (0.593)	0.059 (0.607)	0.025 (0.825)	−0.172 (0.132)	0.004 (0.975)	−0.042 (0.714)	0.024 (0.832)	−0.031 (0.789)	0.062 (0.589)
DASS-21 anxiety	−0.219 (0.054)	−0.113 (0.324)	−0.165 (0.148)	−0.185 (0.105)	−0.161 (0.159)	−0.096 (0.401)	−0.048 (0.679)	−0.114 (0.321)	−0.035 (0.763)
DASS-21 stress	−0.005 (0.967)	−0.003 (0.977)	0.017 (0.882)	−0.074 (0.521)	0.057 (0.618)	0.021 (0.857)	0.039 (0.735)	−0.016 (0.892)	0.062 (0.588)

* p < 0.05; ** p < 0.01 (not corrected for multiple correlations). Significant correlations are in **bold**.

O-LIFE = Oxford-Liverpool Inventory of Feelings and Experiences; SRP-4-SF = Self-Report Psychopathy Scale – Short Form; TriPM = Triarchic Psychopathy Measure; BIS-11 = Barratt Impulsiveness Scale; S-UPPS-P = Impulsive Behavior Scale, Short Version; DASS-21 = Depression, Anxiety, and Stress Scale.

## 3 Results

### 3.1 Sample characteristics

The mean age was 25.96 years (SD = 9.85) with no demographic difference between men (*n* = 25) and women (*n* = 53) (Supplementary Table 1). Native and non-native speakers did not differ in any demographic or self-report measures except anxiety (lower in natives: mean = 12.00, SD = 3.99; non-natives: 14.30, 4.89; *t* = 2.29; df = 76, p = 0.026). Table 1 presents descriptive statistics for all self-report measures.

### 3.2 LDT performance

#### 3.2.1 Accuracy

**Fig. 2:**
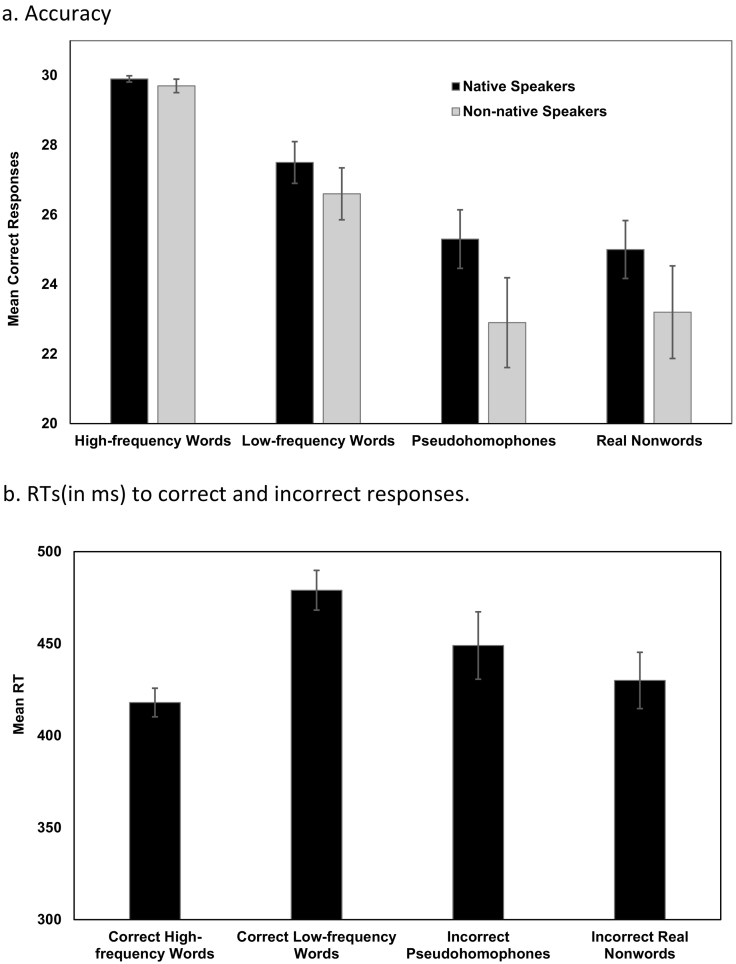
Mean accuracy (2a) for different stimulus-types, and RTs (2b) for correct high and low-frequency words and incorrect pseudohomophones and real nonwords in native (*n* = 42) and non-native speakers (*n* = 36). Error bars display 95% confidence intervals.

There was a main effect of Stimulus-Type [*F*(2.00,153.96) = 99.445, p *<* 0.001, *η*^*2*^_*p*_ = 0.564] (Fig. 2). Participants correctly identified significantly more high-frequency than low-frequency words [*t*(77) = 11.148, p < 0.001], pseudohomophones [*t*(77) = 14.141, p < 0.001], and real nonwords [*t*(77) = 14.700, p *<* 0.001], more low-frequency words than pseudohomophones [*t*(77) = 6.234, p < 0.001] and real nonwords [*t*(77) = 6.449, p < 0.001]; correct pseudohomophones and real nonwords did not differ [*t*(77) = 0.111, p = 0.912]. The main effect of Sex [*F*(1,76) = 0.034, p *=* 0.855] and Sex*Stimulus-Type interaction [*F*(2.01,152.47) = 0.792, p = 0.455] were non-significant.

Language had a significant main effect [*F*(1,76) = 12.290, p = 0.001, *η*^*2*^_*p*_ = 0.139] and interacted with Stimulus-Type [*F*(2.01,152.66) = 3.226, p *=* 0.042, *η*^*2*^_*p*_ = 0.041], indicating that natives were better than non-natives in distinguishing pseudohomophones [*t*(76) = 3.000, p *=* 0.004], and real nonwords [*t*(76) = 2.307, p *=* 0.024] but the groups failed to differ formally in recognition of high-frequency [*t*(76) = 1.965, p = 0*.*053] or low-frequency words [*t*(76) = 1.867, p *=* 0.066] (Table 2). The Sex*Language [*F*(1,76) = 0.773, p *=* 0.382] and Sex*Language*Stimulus-Type interactions [*F*(2.02,149.29) = 0.309, p = 0.736] were non-significant.

#### 3.2.2 RTs

There was a significant main effect of Stimulus-Type for correct words [*F*(1,74) = 240.166, p < 0.001, *η*^*2*^_*p*_ = 0.764] but not for incorrect nonwords [*F*(1,74) = 3.594, p *=* 0.062, *η*^*2*^_*p*_ = 0.046]. Participants were significantly slower when identifying low-frequency than high-frequency words [*t*(77) = 17.316, p < 0.001] and slower when incorrectly identifying pseudohomophones over real nonwords [*t*(77) = 2.440, p = 0.017]. Sex or Language had no significant effect.

3.2.3. LDT Performance: speed-accuracy trade-off.

Longer RTs for incorrect real nonwords correlated with higher real nonword accuracy (*r*_s_ = 0.254, p = 0.025). When examined separately in native and non-native speakers, this was true only for non-natives (non-native: *r*_s_ = 0.490, p *=* 0.002; native: *r*_s_ = 0.052; *Z* *=* 2.05, p = 0.02). Furthermore, only in natives, longer RTs for high-frequency words correlated with their lower accuracy (native: *r*_s_ = −0.395, p = 0.010; non-native: *r*_s_ = 0.118; *Z* *=* 2.27, p = 0.012).

### 3.3 Relationship between LDT performance and psychopathology dimensions

#### 3.3.1 Correlations

Higher Unusual Experiences correlated with lower overall performance (Table 3). Higher psychopathy scores, especially SRP-4-SF Antisocial and TriPM Meanness, also correlated with lower overall performance (Table 3). Higher Antisocial scores correlated with lower word recognition. Higher SRP-4-SF Interpersonal, Affective, Antisocial, and TriPM Meanness correlated with lower correct pseudohomophones recognition. Higher TriPM Boldness and Meanness correlated with lower correct real nonwords recognition. No correlation coefficients in relation to schizotypy or psychopathy differed between native and non-native speakers.

Higher impulsivity correlated with poor LDT performance (Table 3). Specifically, higher S-UPPS-P Sensation Seeking correlated with lower overall performance, and with fewer correct real nonwords. Higher S-UPPS-P Positive Urgency correlated with lower low-frequency words recognition, and higher BIS-11 Motor and Self-Control with lower correct recognition of low-frequency words. For RTs, higher BIS-11 Lack of Perseverance correlated with longer incorrect real-nonword RTs.

Some Impulsivity-LDT correlations were different between native and non-native speakers (Table 4). Specifically, higher BIS-11 Cognitive Instability was associated with more correctly identified low-frequency words in natives only, with significant between-group differences in correlation coefficients (*Z* *=* 2.47, p *=* 0.013). Higher BIS-11 Perseverance correlated with a lower number of correct low-frequency words in non-natives only (between-group difference, Z = 2.5, p = 0.012). Higher BIS-11 Motor and higher S-UPPS-P Positive Urgency correlated with fewer correct low-frequency words in non-natives only (BIS-11 Motor, *Z* *=* 3.22, p = 0.001; S-UPPS-P Positive Urgency, *Z* *=* 2.30, p = 0.021). Overall, in non-natives, BIS-11 Motor impulsivity predicted 30% of the variance in correctly identified low-frequency words [*F*(1,34) = 14.714, p = 0.001, *R*^2^ = 0.302]. In natives, only Cognitive Instability significantly predicted variance (14.7%) in low-frequency words [*F*(1,40) = 6.878, p = 0.012, *R*^2^ = 0.147]. Other measures were excluded as non-significant.

**Table 4:** Relationship between LDT performance and self-reported impulsivity in the native and non-native speakers.

Measure	Overall performance		Correct words high frequency		Correct words low frequency		Correct pseudo-homophones		Correct real nonwords	
	Native	Non-native	Native	Non-native	Native	Non-native	Native	Non-native	Native	Non-native
	*r*_*s*_ (p)	*r*_*s*_ (p)	*r*_*s*_ (p)	*r*_*s*_ (p)	*r*_*s*_ (p)	*r*_*s*_ (p)	*r*_*s*_ (p)	*r*_*s*_ (p)	*r*_*s*_ (p)	*r*_*s*_ (p)
BIS-11 attention	−0.021	0.042	−0.003	−0.237	0.206	0.268	−0.135	0.031	−0.089	−0.172
	(0.896)	(0.806)	(0.983)	(0.164)	(0.190)	(0.114)	(0.395)	(0.858)	(0.576)	(0.317)
BIS-11 cognitive instability	0.038	−0.015	0.034	−0.079	0.451^⁎⁎^	−0.098	−0.182	0.023	−0.027	−0.104
	(0.812)	(0.929)	(0.831)	(0.648)	(0.003)	(0.570)	(0.248)	(0.894)	(0.868)	(0.547)
BIS-11 motor	−0.192	−0.243	−0.212	−0.224	−0.003	−0.644^⁎⁎⁎^	−0.220	0.028	−0.208	−0.109
	(0.223)	(0.153)	(0.177)	(0.189)	(0.984)	(< 0.001)	(0.161)	(0.871)	(0.185)	(0.528)
BIS-11 perseverance	−0.220	0.158	−0.034	0.184	−0.187	0.381^⁎^	0.004	0.005	−0.275	0.044
	(0.162)	(0.358)	(0.831)	(0.281)	(0.235)	(0.022)	(0.980)	(0.977)	(0.078)	(0.799)
BIS-11 self-control	−0.171	−0.121	0.175	−0.247	−0.254	−0.341^⁎^	−0.113	−0.022	−0.116	0.008
	(0.279)	(0.483)	(0.268)	(0.146)	(0.105)	(0.042)	(0.477)	(0.898)	(0.464)	(0.965)
BIS-11 cognitive complexity	0.182	−0.042	0.102	−0.304	−0.226	−0.149	0.245	−0.005	0.235	0.015
	(0.249)	(0.808)	(0.522)	(0.072)	(0.151)	(0.387)	(0.117)	(0.975)	(0.134)	(0.933)
S-UPPS-P negative urgency	−0.196	0.053	−0.040	0.034	0.018	−0.151	−0.182	0.075	−0.264	0.121
	(0.214)	(0.757)	(0.799)	(0.845)	(0.909)	(0.378)	(0.248)	(0.665)	(0.091)	(0.482)
S-UPPS-P lack of perseverance	−0.053	0.062	0.200	0.071	−0.111	−0.156	−0.052	−0.043	0.059	0.260
	(0.740)	(0.721)	(0.204)	(0.681)	(0.484)	(0.362)	(0.742)	(0.803)	(0.709)	(0.126)
S-UPPS-P lack of premeditation	−0.112	−0.007	−0.095	−0.139	0.007	−0.303	−0.256	0.132	−0.041	0.107
	(0.481)	(0.967)	(0.551)	(0.418)	(0.967)	(0.072)	(0.102)	(0.444)	(0.798)	(0.534)
S-UPPS-P sensation	−0.247	−0.352^⁎^	0.067	−0.199	−0.173	−0.217	0.025	−0.266	−0.327^⁎^	−0.310
	(0.115)	(0.035)	(0.672)	(0.245)	(0.274)	(0.204)	(0.875)	(0.117)	(0.035)	(0.066)
S-UPPS-P positive urgency	−0.253	−0.202	−0.165	−0.193	−0.020	−0.511^⁎⁎⁎^	−0.249	−0.028	−0.297	−0.061
	(0.106)	(0.238)	(0.295)	(0.260)	(0.901)	(0.001)	(0.112)	(0.871)	(0.056)	(0.723)

* p < 0.05; ** p < 0.01; *** p < 0.001 (*not corrected for multiple correlations*)*.* Correlations in **bold** are significantly different between native and non-native speakers*.* BIS-11 = Barratt Impulsiveness Scale; S-UPPS-P = Impulsive Behavior Scale, Short Version.

Affective traits did not correlate with performance (Table 3).

#### 3.3.2 The overall model: LDT and psychopathology traits

The stepwise regression model revealed that Meanness, Boldness, and Unusual Experiences predicted over 21% of the overall performance [*F*(3,74) = 6.597, p *=* 0.001, *R*^2^ = 0.211], with Meanness accounting for nearly 12% [F(1,76) Change = 10.238, p *=* 0.002, *R*^2^ Change = 0.119], and Boldness [*F*(1,75) Change = 4.348, p *=* 0.040, *R*^2^ Change = 0.048] and Unusual Experiences [*F*(1,74) Change = 4.128, p = 0.046, *R*^2^ Change = 0.044] accounting for about 4% each. Other traits did not change the predictive value of the overall model. For RTs for incorrect real nonwords, Boldness and BIS-11 Perseverance were entered as predictors, and only Boldness was significant, accounting for 12% of the variance [*F*(1,76) = 3.243, p = 0.002, *R*^*2*^ = 0.122].

## 4 Discussion

As hypothesised, the link between poorer LDT performance and psychopathology-related traits was true for psychopathic traits (Meanness, Boldness) and marginally for positive schizotypy, but not for affective traits. Meanness significantly predicted pseudohomophone and real nonwords accuracy, and Boldness predicted the RTs for incorrect real nonwords. In the overall model, Meanness and Boldness were better predictors of the overall LDT performance than positive schizotypy. Additionally, only in non-native speakers, higher Motor Impulsivity was linked to poorer identification of low-frequency words.

### 4.1 Lexical decision performance: schizotypy versus psychopathy

Meanness (callous aggression and lack of empathy, mostly associated with the affective facet of Psychopathy Checklist-Revised) had the strongest association with LDT performance. Meanness is often elevated in forensic populations (Hare, 2006; Hare and Neumann, 2009) and is associated with criminal behavior whereas Boldness (fearless dominance) is often seen in successful psychopaths (Patrick et al., 2009). Previously, the impulsive-antisocial aspect (similar to TriPM Boldness) was found associated with lower LDT accuracy in highly psychopathic individuals, purportedly caused by reduced processing of changing demands (Heritage and Benning, 2013). Highly psychopathic individuals demonstrate deficits, relative to controls, in processing abstract words and are unable to integrate this information and modulate their behavior accordingly (Kiehl et al., 2004). Also, individuals with higher fearless dominance (Boldness) tend to respond instantaneously which could lead to mistakes in real nonword identification. It is possible that highly psychopathic individuals, especially those with traits associated with criminal behavior, are unable to modulate their responses and poor at integrating various reading skills at once when dealing with more complex lexical information.

In contrast to psychopathy, schizotypy (Unusual Experiences) was less strongly linked to LDT performance (explaining only about 4% of the variance in performance) and did not resemble the relationship seen in schizophrenia. Processes involved in lexical recognition, reading deficits, and dyslexia can be associated with genetic-neuropsychological aspects of schizophrenia as some deficits are also observed in high clinical risk for schizophrenia (Revheim et al., 2014; Whitford et al., 2018). However, normal-to-mildly elevated schizotypal scores without a presence of clinical diagnosis may not necessarily lead to alterations in lexical processing. The deficits in higher schizotypy in language-related tasks can be very subtle, dependent on the tested cohort and specific schizotypy dimensions, or not present at all (Schofield and Mohr, 2014). Furthermore, some of the reading skills deficits seen in schizophrenia may well be explained by medication (de Boer et al., 2020).

### 4.2 Lexical decision, impulsivity and the role of language familiarity

In non-native speakers, higher motor impulsivity was associated with lower accuracy of low-frequency words, but not nonword recognition, suggesting that these individuals may opt for the first interpretation when facing an unfamiliar word and confound it as a nonword; they may “guess” the answer because of poor ability to suppress inadequate vocabulary representations (van der Schoot et al., 2004). Other data also suggest that impulsive individuals process language information less efficiently and often experience problems in processing complex lexical information (De Pascalis et al., 2009; Ku et al., 2020). Unexpectedly, in native speakers, Cognitive Instability, which captures impulsive, quickly changing thoughts (Patton et al., 1995), was associated with better identification of low-frequency words, possibly by helping them shift quickly between different lexical representations and select the correct one (with good knowledge of the language).

### 4.3 Implications and limitations

Our present findings show that elevated psychopathic traits and higher motor impulsivity in combination with non-native language proficiency are associated with poor lexical recognition. Considering previous findings of impaired reading skills in patients with psychopathy and/or a history of violence (Vanova et al., 2021), our results suggest the existence of a continuum of reading skill deficits related to elevated psychopathic traits and have implications for future research adopting a dimensional approach to psychopathology. Future research could establish whether the mechanisms underlying psychopathy/schizotypy-lexical recognition association in the normative population are shared with those underlying poor reading skills in clinical populations, what it means in terms of vulnerability to dyslexia, and clarify the roles of specific symptoms and illness-related factors (e.g., medication) (de Boer et al., 2020). People with high psychopathy in forensic and non-forensic populations show impairments in various reading skills, including lexical recognition, and a high prevalence of dyslexia (Brites et al., 2015; Daderman et al., 2004; Selenius et al., 2006). Especially vulnerable are non-native speakers from an immigrant background (Svensson et al., 2015), a factor associated with a risk for schizophrenia (Selten et al., 2007). Vulnerability to dyslexia can negatively influence their socio-economic status and academic achievements (Hemphill and Tivnan, 2008). Our findings on psychopathic traits could help to better understand the cognitive challenges associated with these traits, their links with dyslexia, even in educated populations.

This study, however, had limitations, including (i) a relatively small sample size and limited range of schizotypal and psychopathy scores in the sample, (ii) unexpectedly, an influence of language familiarly in impulsivity-LDT association, (iii) use of a one-choice variant LDT (i.e., no RTs for correct nonwords), and (iv) no correction for multiple testing which could lead to Type-I error. Thus, our findings should be considered preliminary until replicated in future studies with larger samples and other LDT variants. Furthermore, this was a correlational study, thus, we cannot infer causation.

## 5 Conclusions

We found that psychopathic traits show stronger negative associations with lexical recognition than schizotypal traits, and impulsivity may differentially affect performance depending on language familiarity. There is, however, a need to replicate these findings, especially the influence of language familiarity in the impulsivity-performance relationship.

## Sources of funding

This research received no specific grant from any funding agency, commercial or not-for-profit sectors. Martina Vanova and Luke Aldridge-Waddon were supported by 10.13039/501100007914Brunel University London College of Health, Medicine, and Life Sciences Doctoral Scholarships. Leonie Elbers was supported by the Erasmus Mobility programme.

## CRediT authorship contribution statement

**Martina Vanova:** Conceptualization; Formal analysis; Data curation; Investigation; Methodology; Project administration; Visualization; Writing - original draft; Writing - review & editing. **Luke Aldrige-Waddon:** Project administration; Writing - review & editing. **Ben Jennings:** Formal analysis; Supervision; Writing - review & editing. **Leonie Elbers:** Project administration; Writing - review & editing. **Ignazio Puzzo:** Supervision; Writing - review & editing. **Veena Kumari:** Conceptualization, Methodology, Formal analysis, Resources, Writing - review & editing, Supervision, Funding acquisition.

## Declaration of competing interest

None.

## Appendix A Supplementary data

Supplementary tables Image 1
