# Supplementary material for: Clarifying the roles of schizotypy and psychopathic traits in lexical decision performance
Source: Schizophr Res Cogn. 2021 Nov 16;27:100224. doi: 10.1016/j.scog.2021.100224 (PMC8605281; doi:10.1016/j.scog.2021.100224)
Supplement: Supplementary file 1 — Supplementary tables [file mmc1.docx]

**Supplementary Table 1.** Full sample characteristics.

| Variable/*N* | Males  (n= 25) | Females  (n= 53) | Overall sample  (n= 78) |
| --- | --- | --- | --- |
| Age (Mean [±*SD*]) | 25.96 (9.85) | 22.74 (4.48) | 23.68 (7.73) |
| Language (Number [%]) |  |  |  |
| English | 16 (64%) | 26 (49.1%) | 44 (55.0%) |
| Other | 9 (36%) | 27 (50.9%) | 36 (45.0%) |
| Ethnicity (Number [%]) |  |  |  |
| White | 14 (56.0%) | 20 (37.7%) | 36 (45.0%) |
| Asian/Pacific Islander | 6 (24.0%) | 19 (35.8%) | 25 (31.3%) |
| Black/African American | 0 | 5 (9.45) | 5 (6.3%) |
| Hispanic/Latino | 0 | 1 (1.9%) | 1 (1.3%) |
| Other | 5 (20.0%) | 8 (15.1%) | 13 (16.3%) |
| Handedness (Number [%]) |  |  |  |
| Right | 23 (92.0%) | 49 (92.5%) | 73 (91.3%) |
| Left | 2 (8.0%) | 4 (7.5%) | 7 (8.8%) |
| Education (Number [%]) |  |  |  |
| Higher Degree | 4 (16.0%) | 9 (17.0%) | 13 (16.3%) |
| First Degree | 10 (40.0%) | 14 (26.4%) | 24 (30.0%) |
| Teaching Qualification | 1 (4.0%) | 0 | 1 (1.3%) |
| Other Higher Qualification | 0 | 4 (7.5%) | 4 (5.0%) |
| GCE A Level in 2+ | 6 (24.0%) | 21 (39.6%) | 29 (36.3%) |
| GCE A Level in 1 | 1 (4.0%) | 0 | 1 (1.3%) |
| GCSE/O Level in 5 | 3 (12.0%) | 3 (5.7%) | 6 (7.5%) |
| GCSE/O Level in 1-4 | 0 | 1 (1.9%) | 1 (1.3%) |
| CSE below 1/GCSE below Grade C | 0 | 1 (1.9%) | 1 (1.3%) |

**Supplementary Table 2.** Spearman inter-correlations (*p* values in italics) between psychopathology traits in the entire sample (*n*=78).

|  | Schizotypy | | | Psychopathy | | | | | | | | | Impulsivity | | | | | | | Affective | | |
| --- | --- | --- | --- | --- | --- | --- | --- | --- | --- | --- | --- | --- | --- | --- | --- | --- | --- | --- | --- | --- | --- | --- |
|  | 2 | 3 | 4 | 5 | 6 | 7 | 8 | 9 | 10 | 11 | 12 | 13 | 14 | 15 | 16 | 17 | 18 | 19 | 20 | 21 | 22 | 23 |
| 1. O-LIFE Unusual Experiences | .560^**^ | .206 | .497^**^ | .185 | .491^**^ | .284^*^ | .076 | .239^*^ | .078 | .295^**^ | .116 | .478^**^ | -.126 | .359^**^ | .258^*^ | .359^**^ | .275^*^ | .163 | .188 | .256^*^ | .585^**^ | .353^**^ |
|  | *<.001* | *.070* | *<.001* | *.105* | *<.001* | *.012* | *.506* | *.035* | *.499* | *.009* | *.313* | *<.001* | *.271* | *.001* | *.022* | *.001* | *.015* | *.155* | *.099* | *.024* | *<.001* | *.002* |
| 2. O-LIFE Cognitive Distortions |  | .361^**^ | .387^**^ | .450^**^ | .510^**^ | .126 | .366^**^ | .405^**^ | .220 | .391^**^ | -.240^*^ | .318^**^ | -.613^**^ | .400^**^ | .072 | .195 | .087 | .002 | .061 | .553^**^ | .615^**^ | .614^**^ |
|  |  | *.001* | *<.001* | *<.001* | *<.001* | *.270* | *.001* | *<.001* | *.053* | *<.001* | *.035* | *.005* | *<.001* | *<.001* | *.533* | *.088* | *.448* | *.988* | *.593* | *<.001* | *<.001* | *<.001* |
| 3. O-LIFE Introvertive Anhedonia |  |  | .007 | .101 | .082 | -.236^*^ | .019 | -.033 | .084 | .085 | -.323^**^ | -.078 | -.411^**^ | .136 | .213 | .085 | .272^*^ | -.040 | .099 | .399^**^ | .301^**^ | .327^**^ |
|  |  |  | *.955* | *.379* | *.476* | *.037* | *.869* | *.775* | *.466* | *.458* | *.004* | *.498* | *.000* | *.235* | *.061* | *.460* | *.016* | *.731* | *.388* | *.000* | *.007* | *.004* |
| 4. O-LIFE Impulsive Nonconform. |  |  |  | .358^**^ | .437^**^ | .549^**^ | .268^*^ | .489^**^ | .205 | .499^**^ | .225^*^ | .520^**^ | -.023 | .592^**^ | .481^**^ | .514^**^ | .185 | .436^**^ | .337^**^ | .296^**^ | .262^*^ | .361^**^ |
|  |  |  |  | *.001* | *<.001* | *<.001* | *.018* | *<.001* | *.072* | *<.001* | *.048* | *<.001* | *.838* | *<.001* | *<.001* | *<.001* | *.104* | *<.001* | *.003* | *.009* | *.021* | *.001* |
| 5. BIS-11 Attention |  |  |  |  | .424^**^ | .300^**^ | .283^*^ | .474^**^ | .233^*^ | .113 | -.013 | .161 | -.210 | .373^**^ | .329^**^ | .273^*^ | .202 | .308^**^ | .086 | .342^**^ | .274^*^ | .168 |
|  |  |  |  |  | *<.001* | *.008* | *.012* | *<.001* | *.040* | *.324* | *.912* | *.160* | *.065* | *.001* | *.003* | *.016* | *.077* | *.006* | *.453* | *.002* | *.015* | *.142* |
| 6. BIS-11 Cognitive Instability |  |  |  |  |  | .226^*^ | .237^*^ | .234^*^ | -.101 | .262^*^ | .053 | .346^**^ | -.182 | .351^**^ | .294^**^ | .370^**^ | .245^*^ | .335^**^ | .106 | .218 | .334^**^ | .333^**^ |
|  |  |  |  |  |  | *.047* | *.037* | *.039* | *.379* | *.020* | *.644* | *.002* | *.111* | *.002* | *.009* | *.001* | *.030* | *.003* | *.356* | *.055* | *.003* | *.003* |
| 7. BIS-11 Motor |  |  |  |  |  |  | .055 | .515^**^ | .223^*^ | .348^**^ | .330^**^ | .610^**^ | .267^*^ | .526^**^ | .380^**^ | .457^**^ | .294^**^ | .467^**^ | .316^**^ | .082 | .137 | .084 |
|  |  |  |  |  |  |  | *.633* | *<.001* | *.049* | *.002* | *.003* | *<.001* | *.018* | *<.001* | *.001* | *<.001* | *.009* | *<.001* | *.005* | *.478* | *.233* | *.467* |
| 8. BIS-11 Perseverance |  |  |  |  |  |  |  | .459^**^ | .293^**^ | .390^**^ | .019 | .211 | -.254^*^ | .230^*^ | .051 | .020 | -.046 | .120 | .139 | .375^**^ | .153 | .407^**^ |
|  |  |  |  |  |  |  |  | *<.001* | *.009* | *<.001* | *.867* | *.063* | *.025* | *.043* | *.660* | *.863* | *.692* | *.295* | *.224* | *.001* | *.181* | *<.001* |
| 9. BIS-11 Self Control |  |  |  |  |  |  |  |  | .518^**^ | .331^**^ | .217 | .444^**^ | -.148 | .503^**^ | .320^**^ | .136 | .013 | .337^**^ | .139 | .293^**^ | .199 | .260^*^ |
|  |  |  |  |  |  |  |  |  | *<.001* | *.003* | *.056* | *<.001* | *.196* | *<.001* | *.004* | *.236* | *.907* | *.003* | *.225* | *.009* | *.081* | *.022* |
| 10. BIS-11 Cognitive Complexity |  |  |  |  |  |  |  |  |  | .126 | -.030 | .209 | -.262^*^ | .363^**^ | .059 | .015 | -.097 | .066 | .017 | .174 | .154 | .279^*^ |
|  |  |  |  |  |  |  |  |  |  | *.272* | *.793* | *.066* | *.021* | *.001* | *.606* | *.894* | *.398* | *.567* | *.882* | *.129* | *.179* | *.013* |
| 11. S-UPPS-P Negative Urgency |  |  |  |  |  |  |  |  |  |  | .063 | .582^**^ | -.232^*^ | .535^**^ | .203 | .249^*^ | .110 | .258^*^ | .334^**^ | .415^**^ | .310^**^ | .413^**^ |
|  |  |  |  |  |  |  |  |  |  |  | *.584* | *<.001* | *.041* | *<.001* | *.074* | *.028* | *.336* | *.023* | *.003* | *<.001* | *.006* | *<.001* |
| 12. S-UPPS-P Sensation Seeking |  |  |  |  |  |  |  |  |  |  |  | .342^**^ | .484^**^ | .148 | .226^*^ | .189 | .161 | .400^**^ | .080 | -.156 | -.101 | -.039 |
|  |  |  |  |  |  |  |  |  |  |  |  | *.002* | *<.001* | *.197* | *.046* | *.098* | *.160* | *<.001* | *.488* | *.173* | *.378* | *.732* |
| 13. S-UPPS-P Positive Urgency |  |  |  |  |  |  |  |  |  |  |  |  | .103 | .606^**^ | .369^**^ | .389^**^ | .295^**^ | .465^**^ | .330^**^ | .230^*^ | .300^**^ | .270^*^ |
|  |  |  |  |  |  |  |  |  |  |  |  |  | *.368* | *<.001* | *.001* | *<.001* | *.009* | *<.001* | *.003* | *.043* | *.008* | *.017* |
| 14. TriPM Boldness |  |  |  |  |  |  |  |  |  |  |  |  |  | -.109 | .084 | .008 | .098 | .163 | -.006 | -.444^**^ | -.359^**^ | -.464^**^ |
|  |  |  |  |  |  |  |  |  |  |  |  |  |  | *.344* | *.467* | *.947* | *.391* | *.154* | *.956* | *<.001* | *.001* | *<.001* |
| 15. TriPM Disinhibition |  |  |  |  |  |  |  |  |  |  |  |  |  |  | .518^**^ | .450^**^ | .379^**^ | .553^**^ | .329^**^ | .449^**^ | .383^**^ | .423^**^ |
|  |  |  |  |  |  |  |  |  |  |  |  |  |  |  | *<.001* | *<.001* | *.001* | *<.001* | *.003* | *<.001* | *.001* | *<.001* |
| 16. TriPM Meanness |  |  |  |  |  |  |  |  |  |  |  |  |  |  |  | .590^**^ | .574^**^ | .676^**^ | .360^**^ | .227^*^ | .230^*^ | .232^*^ |
|  |  |  |  |  |  |  |  |  |  |  |  |  |  |  |  | *<.001* | *<.001* | *<.001* | *.001* | *.046* | *.042* | *.041* |
| 17. SRP-4-SF Interpersonal |  |  |  |  |  |  |  |  |  |  |  |  |  |  |  |  | .663^**^ | .583^**^ | .263^*^ | .268^*^ | .242^*^ | .290^*^ |
|  |  |  |  |  |  |  |  |  |  |  |  |  |  |  |  |  | *<.001* | *<.001* | *.020* | *.018* | *.033* | *.010* |
| 18. SRP-4-SF Affective |  |  |  |  |  |  |  |  |  |  |  |  |  |  |  |  |  | .551^**^ | .215 | .323^**^ | .270^*^ | .197 |
|  |  |  |  |  |  |  |  |  |  |  |  |  |  |  |  |  |  | *<.001* | *.059* | *.004* | *.017* | *.084* |
| 19. SRP-4-SF Lifestyle |  |  |  |  |  |  |  |  |  |  |  |  |  |  |  |  |  |  | .357^**^ | .152 | .087 | .160 |
|  |  |  |  |  |  |  |  |  |  |  |  |  |  |  |  |  |  |  | *.001* | *.183* | *.448* | *.162* |
| 20. SRP-4-SF Antisocial |  |  |  |  |  |  |  |  |  |  |  |  |  |  |  |  |  |  |  | .189 | .240^*^ | .244^*^ |
|  |  |  |  |  |  |  |  |  |  |  |  |  |  |  |  |  |  |  |  | *.098* | *.034* | *.031* |
| 21. DASS-21 Depression |  |  |  |  |  |  |  |  |  |  |  |  |  |  |  |  |  |  |  |  | .577^**^ | .660^**^ |
|  |  |  |  |  |  |  |  |  |  |  |  |  |  |  |  |  |  |  |  |  | *<.001* | *<.001* |
| 22. DASS-21 Anxiety |  |  |  |  |  |  |  |  |  |  |  |  |  |  |  |  |  |  |  |  |  | .591^**^ |
|  |  |  |  |  |  |  |  |  |  |  |  |  |  |  |  |  |  |  |  |  |  | *<.001* |

** p < .05; ** p < .01* (*not corrected for multiple correlations*)

O-LIFE = Oxford-Liverpool Inventory of Feelings and Experiences; SRP-4-SF = Self-Report Psychopathy Scale – Short Form; TriPM = Triarchic Psychopathy Measure; BIS-11 = Barratt Impulsiveness Scale; S-UPPS-P = Impulsive Behavior Scale, Short Version; DASS-21 = Depression, Anxiety, and Stress Scale.
